# Dietary or supplemental fermentable fiber intake reduces the presence of *Clostridium XI* in mouse intestinal microbiota: The importance of higher fecal bacterial load and density

**DOI:** 10.1371/journal.pone.0205055

**Published:** 2018-10-02

**Authors:** Wei Zheng, Kairui Wang, Yijun Sun, Shiu-Ming Kuo

**Affiliations:** 1 Department of Computer Science and Engineering, University at Buffalo, Buffalo, NY, United States of America; 2 Department of Exercise and Nutrition Sciences, University at Buffalo, Buffalo, NY, United States of America; 3 Department of Microbiology and Immunology, University at Buffalo, Buffalo, NY, United States of America; 4 Department of Biostatistics, University at Buffalo, Buffalo, NY, United States of America; University of Florida, UNITED STATES

## Abstract

**Objectives:**

*Clostridium difficile* infection is a public health concern. *C*. *difficile* was found in healthy human intestine as a member of *Clostridium XI*. Because soluble fermentable fiber ingestion affects intestinal microbiota, we used fiber-containing diets to determine the intestinal microbial condition that could reduce the presence of *Clostridium XI*.

**Methods:**

Newly weaned male mice were assigned to three published diets: Control AIN-93G purified diet with only poorly fermented cellulose; Control plus 5% purified fermentable fiber inulin; Chow with wheat, soybean and corn that provide a mixture of unpurified dietary fibers. Methods were developed to quantify 24-hour fecal microbial load and microbial DNA density. The relative abundance of bacterial genera and the bacterial diversity were determined through 16S rRNA sequence-based fecal microbiota analysis.

**Results:**

Mice adjusted food intake to maintain the same energy intake and body weight under these three moderate-fat (7% w:w) diets. Chow-feeding led to higher food intake but also higher 24-h fecal output. Chow-feeding and 1–8 wk ingestion of inulin-supplemented diet increased daily fecal microbial load and density along with lowering the prevalence of *Clostridium XI* to undetectable. *Clostridium XI* remained undetectable until 4 weeks after the termination of inulin-supplemented diet. Fermentable fiber intake did not consistently increase probiotic genera such as *Bifidobacterium* or *Lactobacillus*. Chow feeding, but not inulin supplementation, increased the bacterial diversity.

**Conclusions:**

Increase fecal microbial load/density upon fermentable fiber ingestion is associated with a lower and eventually undetectable presence of *Clostridium XI*. Higher bacterial diversity or abundance of particular genera is not apparently essential. Future studies are needed to see whether this observation can be translated into the reduction of *C*. *difficile* at the species level in at-risk populations.

## Introduction

*Clostridium difficile* infection is a major burden in US health care system and can lead to sepsis and death [1, 2]. The pathogenic *C*. *difficile* species belong to the genus *Clostridium XI* [3, 4] which is a member of the intestinal microbiota. Patients with *C*. *difficile* infection showed a higher prevalence of *Clostridium XI* [5]. Asymptomatic *C*. *difficile* colonization was observed in healthy individuals and their *Clostridium XI* level was lower than those with active *C*. *difficile* infection [5, 6]. The objective of the study is to use controlled fiber feeding to determine intestinal microbial factors that reduce the presence of *Clostridium XI* in the large intestine.

Dietary fiber family is made of nondigestible carbohydrates with vastly different sizes, structures and bacterial fermentabilities. In this study, poorly fermentable cellulose is the only fiber included in the Control AIN-93G diet [7]. Purified inulin from chicory [8, 9] is an approved fructan supplement and widely used soluble fermentable fiber by food manufacturers. We have formulated inulin-supplemented AIN-93G diet [10] and found bile acid-modifying [10] and anti-inflammatory [11] effects in mice studies. The ability of inulin to modify intestinal microbiota human and other mammalian species was recently reviewed [12, 13]. Other isolated/purified dietary plant fibers have also been found to modify intestinal microbiota [12, 13]. However, most studies did not address the effect of unpurified fiber as a part of normal diet. To address the collective effect of multiple unpurified fibers, a group of chow-fed mice was included in this study. Rodent chow commonly used in animal facilities contains fiber (soluble and insoluble) from its ingredients, wheat, soybean and corn [14]. It mimics the plant-enriched dietary choice among humans.

One unique aspect of the mouse study is the collection and processing of 24-hr feces. The parallel food intake, fecal collection and body weight measurement along with microbiome analysis can help to address the possible link if any between body weight and intestinal microbiome. More importantly, 24-h fecal recovery provides information on the daily total fecal bacterial load and the fecal bacterial density. These quantitative characteristics of intestinal microbes are complementary to the relative abundance of bacteria genera obtained from operational taxonomic units (OTU)-based microbiome analysis. A few previous studies have reported higher fecal microbial density after soluble fiber supplementation [12]. An increase in the soluble fiber/insoluble fiber ratio in the diet led to more fermentation by-products in dogs [15]. However, these studies did not examine the prevalence of *C*. *difficile* or *Clostridium XI* genus. A major risk factor for *C*. *difficile* infection is the antibiotic exposure [16]. Because antibiotics often alter intestinal microbiota, colonization resistance of healthy intestinal microbiota against *C*. *difficile* was proposed [17]. While the contribution of particular microbial genera especially those with probiotic species was usually emphasized [17], we hypothesized that fecal bacterial load and density could also play a role in the colonization resistance. We tested the hypothesis by performing fecal bacterial load, microbial density and microbiota analysis under the three diets described above.

## Materials and methods

### Animal care and sample collection

The protocols for mouse care and handling were approved by the Institutional Animal Care and Use Committee of University at Buffalo. Newly weaned murine pathogen-free inbred C57BL/6NTac mice from a single facility of Taconic Biosciences were used. To avoid the effect of hormonal changes during estrus cycling, only male mice were used. Upon arrival to the conventional animal facility (74°F, 12-h light/12-h dark), they were assigned to one of the three diets: AIN-93G diet containing 5% (wt:wt) poorly fermented insoluble cellulose as the fiber source (Control) [7]; AIN-93G diet supplemented with 5% (wt:wt) soluble and fermentable fiber, purified inulin (Inulin) [10]; rodent chow (Teklad 2018SX, Envigo) with wheat, corn and soybean (Chow). Chow contains 14.7% neutral detergent fiber and unpurified fermentable fiber including inulin [9, 14]. Experimental mice had no restriction on food intake and were given free access to tap water. Laboratory grade Sani-chip (Teklad 7090, Envigo) was used as the bedding material. The same types of plastic cage and bedding material were used before, during and after the fecal collection. To tightly control the fiber intake, no natural fiber-containing cage enrichment was provided.

To measure the absolute changes in the microbial quantity, we developed a method to collect 24-h feces from individual mouse, which allows us to quantify the daily fecal microbial output and daily average microbial density in feces. Fecal microbial composition was shown to be stable during 24 h room temperature storage [18] although nucleotides from exfoliated cells of the gastrointestinal tract degrade quickly [19]. All mice in this study were individually housed and fecal collection was performed at the end of 12-h light cycle. At 24 h before the fecal collection, each mouse was transferred to a clean cage with fresh bedding as part of the weekly cage change. Fecal collection was done while each mouse was transiently removed from the cages for the weekly body weight and food intake measurements. There was no sign of undue stress beyond the normal cage change and health monitoring. No animal injury or loss was observed from the dietary treatment or the fecal collection procedure. To preserve microbial integrity, feces collected into pre-weighed vials were used immediately for DNA extraction or stored in -80°C and used within 6 mo [18]. Mice were killed at the end of dietary treatment. Some 8-wk Inulin-diet mice were switched to the Control diet for another 4 weeks of fecal collection and then killed. Cecal content was collected from the dissected cecum after mice were killed by cervical dislocation at the end of the 12-h light cycle. Cecal content was used for DNA extraction immediately. The protocol for mouse 24-hr feces and cecal content collection has been deposited in Protocols.io for public access (dx.doi.org/10.17504/protocols.io.te7ejhn).

### Bacterial DNA extraction and characterization by PCR

The entire 24-h feces was used for DNA extraction because randomly sampled small quantity of human stool was found not to accurately represent the entire feces in microbial composition [20]. Mouse fecal pellets we collected also varied in appearance (unpublished observation). Cecal DNA was also extracted from the entire cecal content. The samples collected in the same day were generally extracted at the same time. Ground glass pestle and vessel were used for the homogenization of feces after the feces have been hydrated and mashed in ice-cold 0.9% NaCl solution for 30 min. Cecal content was homogenized in ice-cold 0.9% NaCl solution using Kontes kit (#749520) with Kontes pellet pestle motor (#749540). After homogenization, the complete release of microorganism from the fecal and cecal mass was confirmed under the microscope. The homogenized fecal and cecal samples were centrifuged at 900 x g, at 4°C for 5 min to remove large particles including undigested food and intact mouse gastrointestinal cells, if any. The OD_650_ of the supernatant was measured to estimate the total fecal microbial recovery before DNA extraction [21]. The protocols for fecal and cecal bacteria DNA extraction have been deposited in Protocols.io for public accesss (dx.doi.org/10.17504/protocols.io.tfyejpw and dx.doi.org/10.17504/protocols.io.tfeejje).

The supernatant was then centrifuged at a higher speed (15,000 x g, 4 min) to separate bacterial pellets from degraded mouse DNA and other small cellular remnants. Wizard genomic DNA purification kit (Promega Corp. Madison, WI) was used with published modifications in bacterial lysis [22, 23] to extract RNA-free large genomic DNA from the bacterial pellets. OD reading at 260 nm was used to calculate fecal DNA recovery. To ensure the effectiveness of our multiple steps in separating mouse DNA from the bacterial DNA, we performed PCR analysis of mouse genes in the extracted fecal DNA to validate the low mouse DNA contamination. Published PCR primers for three mouse genes, insulin-like growth factor 2, IL10 and sodium-dependent vitamin C transporter 2, were used and mouse tissue DNA that we have reported was used as the mouse DNA standard [24, 25]. For the purpose of quality control in the microbiota analysis and in qPCR quantification, DNA was also extracted from a mixture of known concentrations of bacteria including *Bifidobacterium lactis*, *B*. *breve*, *B*. *longum*, *Lactobacillus acidphilus*, *L*. *casei*, *L*. *plantarum*, *L*. *paracasei*, *L*. *salivarius*, *L*. *rhamnosus*, and *L*. *bulgaricus* (Ultimate Flora, ReNew Life Formula, Palm Harbor, FL) and used as the bacterial DNA standard.

### Sequencing and microbiome analysis

Fecal and cecal DNA samples were analyzed using Illumina MiSeq System in UB Next-Gen Sequencing Core Facility (300-cycle paired end) following the manufacturer’s protocol. Individuals blinded to the dietary treatment performed fecal DNA amplification, sequencing and microbiome data analyses. Technical duplicates of DNA samples and bacterial DNA standard were included at different locations during the amplification and sequencing. Illumina V1-V3 primer pair for 16S-rRNA [26] was used for the first run of sequencing. We found that V1-V3 primer pair could amplify the 16S-rRNA gene of *Lactobacillus* but not that of *Bifidobacterium* based on the results from the bacterial DNA standard. Illumina V3-V4 primer pair for 16S-rRNA was then used for the second run of sequencing [26]. V3-V4 primer pair amplified DNA standard effectively and microbiota analysis yielded *Bifidobacterium* and *Lactobacillus* at the expected prevalence.

All the raw data of MiSeq sequencing results were stored as FASTQ [27] format files. They are available as SRA accession# PRJNA491464 and PRJNA491467 in the NCBI website. Pair-end reads were joined by using PEAR V0.9.6 [28]. Samples with low reads (less than the mean minus 2 SD of the run) were first rejected. Quality filtering was then performed to remove low quality reads by using FASTX-Toolkit V0.0.13 (http://hannonlab.cshl.edu/fastx_toolkit). A merged read with 90% of its bases have Pred quality scores [29] higher or equal to Q30 was considered as a good read and retained for downstream analyses. The samples used for the analyses have an average read of 101,197 and 76,302, respectively, for the run 1 and 2 of sequencing. From the accepted samples, we performed closed-reference OTU picking through BLAST [30] search against the Ribosomal Database Project database [31] with stringent criteria: the identity percentage >97% and the length of the aligned region >97% of the total length. DESeq2 [32] was used to determine which taxonomic units were significantly different between groups (p<0.05). Benjamini-Hochberg method [33] was then applied to control the false discovery rate. Genus level is the lowest taxonomic rank available in Ribosomal Database Project database. Compiling the top five most abundant genera from each sample generated the stacking bar graphs of major genera. The alpha-diversity analysis was performed using the QIIME [34] pipeline. The rarefaction curves show the number of observed OTUs across a range of progressively deeper simulated sequencing depths.

### qPCR analysis of fecal DNA

Because Illumina V1-V3 primer pair could not amplify the *Bifidobacterium* 16S-rRNA sequence, qPCR was also performed on fecal and cecal DNA samples using other *Bifidobacterium*-specific primers [35, 36]. SsoAdvanced Universal SYBR Green Supermix (Bio-Rad Laboratories Inc., Hercules, CA, USA) was used for PCR reaction in iCycler iQ PCR machine (Bio-Rad Laboratories Inc). The Cq was found to be linear to the amount of input *Bifidobacterium* DNA standard from 10^−8^ to 10^−3^ g/L. Two sets of qPCR primers, BgenusA [35] and BgenusB [36], amplified our fecal and cecal samples equally well and the qPCR results from these two primer sets were highly correlated (y (BgenusB) = 6.9 +1.05 x (BgenusA), R^2^ = 0.941, N = 112) (**S1 Fig**). The amount of *Bifidobacterium* DNA in the cecal samples and the corresponding fecal samples collected at the same day showed non-linear positive correlation (S1 Fig).

### Statistical analysis

Seven to eight mice were used for each treatment group, Control, Inulin and Chow. Previous fiber studies on C57BL/6 mice found 5–8 mice per group sufficient in detecting the treatment effects of Inulin diet [10, 11]. For the analysis of food intake, body weight, 24-h fecal weight as well as daily fecal microbial and DNA recovery at each time point, Dunnett’s Multiple Comparison Test [37] was used to test two non-orthogonal contrasts from the three groups at each time point: Inulin vs. Control and Chow vs. Control. The test provides a single step control of Type I error [37]. Significant difference was concluded when p<0.05.

## Results

**Fig 1:**
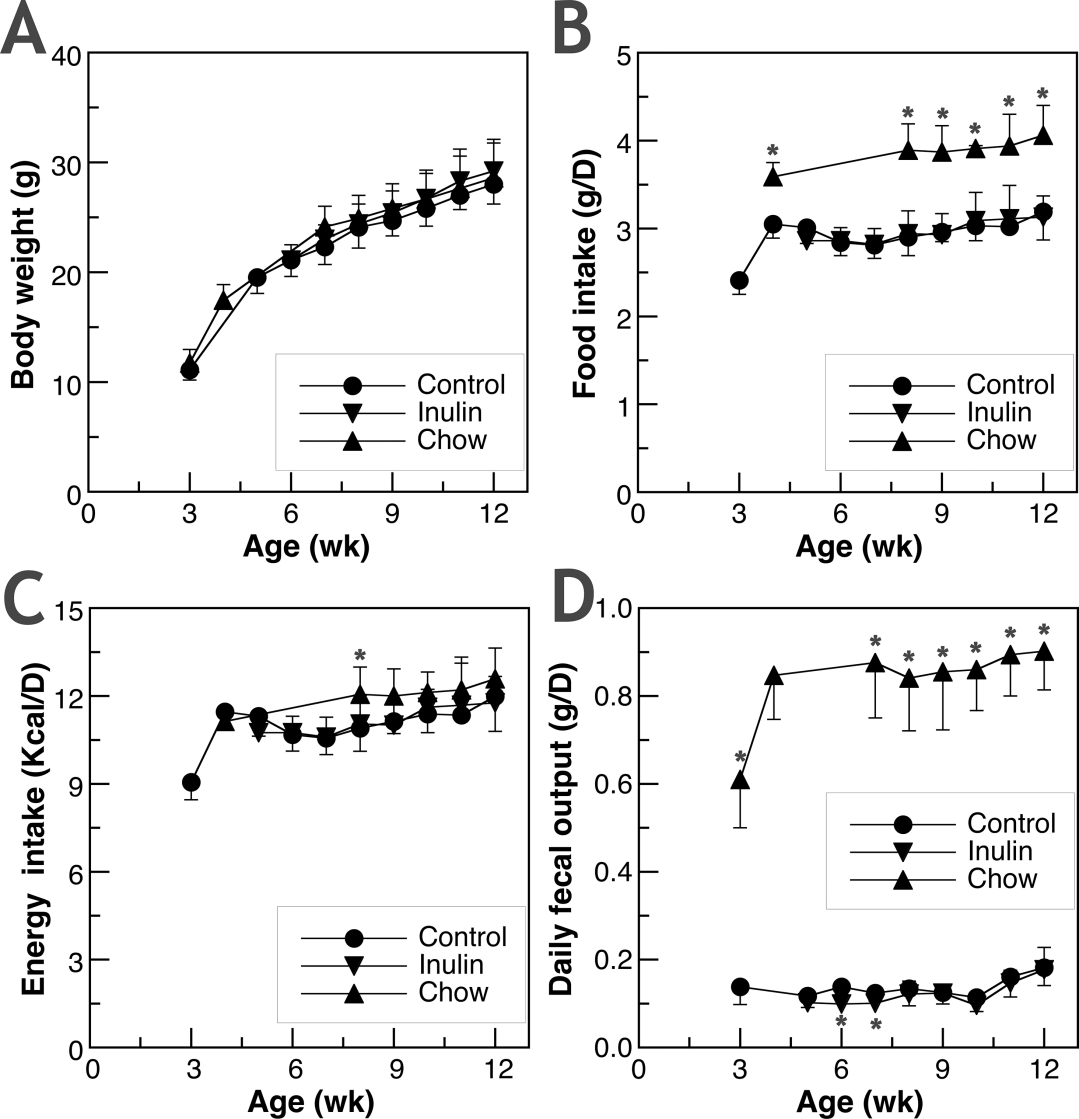
**The effect of inulin supplementation and unpurified dietary fiber on (A) body weight, (B) daily food intake, (C) daily energy intake, and (D) 24-hr fecal output.** Comparisons were made between the newly weaned mice given AIN-93G diet (Control) and those given AIN-93G diet supplemented with 5% inulin (Inulin); as well as between the newly weaned mice given Control and those given wheat, soy, and corn-based rodent chow (Chow). Values shown are mean±SD of 7–8 mice in each group. Data points of Inulin or Chow groups with * are significantly different from that of the Control group at the same age (p<0.05). The energy contents of Control and Inulin were the same at 3,759 Kcal/Kg while Chow had only 3,100 Kcal/Kg.

Food intake and body weight were monitored to determine the effect of fiber on growth. The post-weaning weight gain, daily food intake, and daily energy intake of male C57BL/6 mice given three different diets, Control, Inulin and Chow, are shown in **Fig 1**.

The three different diets, Control, Inulin and Chow, led to similar weight gain (Fig 1A). Chow-fed mice had significant higher daily food intake at all time points (Fig 1B) but the total daily energy intake was mostly not different among three groups (Fig 1C). The 24-h fecal output shown in Fig 1D examines the relationship between fiber intake and fecal bulk. Chow-feeding increased daily fecal output while Inulin-feeding led to similar or slightly lower daily fecal output compared to the Control group. Of all data points from the Chow-fed mice, there is a significant positive linear correlation between daily food intake (or daily neutral detergent fiber intake) and fecal output (**S2 Fig**).

**Fig 2:**
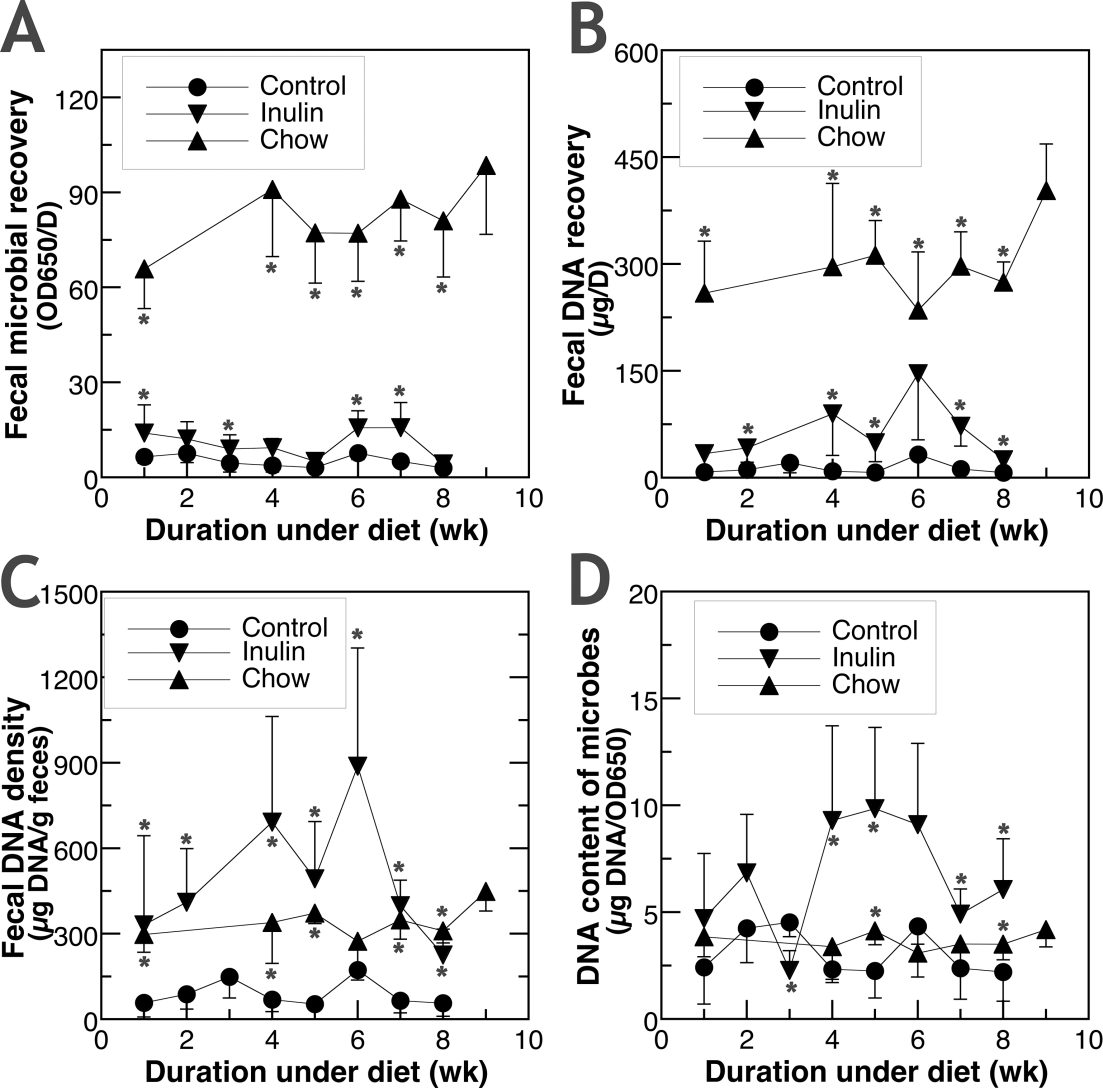
**The effect of inulin supplementation and unpurified dietary fiber on (A) daily fecal microbial recovery, (B) daily fecal DNA recovery, (C) fecal DNA density, and (D) DNA content of microbes.** Comparisons were made between the newly weaned mice given AIN-93G diet (Control) and those given AIN-93G diet supplemented with 5% inulin (Inulin); as well as between the mice given Control and those given wheat, soy, and corn-based rodent chow (Chow). Values shown are mean±SD of 4–8 mice in each group. Data points of Inulin or Chow groups with * are significantly different from that of the respective Control group after the same duration of dietary treatment (p<0.05).

We processed the entire 24-hr feces and used two methods to estimate fecal microbial amount: OD650 of the fecal supernatant after the removal of food remnants and gastrointestinal cells; and OD260 of high-molecular weight DNA extracted from the pelleted fecal supernatant. Using our method to collect and process feces, PCR reactions revealed that mouse DNA accounted for less than 0.5% of the fecal DNA we extracted. **Fig 2** included the results on daily total OD650, total μg fecal DNA, as well as μg fecal DNA normalized by fecal weight and OD650.

Fecal daily total microbial recovery, estimated either by OD650 of fecal supernatant (Fig 2A) or μg DNA extracted (Fig 2B), was significant higher in Inulin and especially Chow groups compared to the Control throughout the duration of dietary treatment. Feeding inulin or Chow with a mixture of unpurified dietary fiber both led to a higher fecal microbial DNA density as well (Fig 2C).

When total fecal DNA was divided by OD650 (total amount of microbes) to determine the DNA content of microbes, the three groups were much more similar (Fig 2D). Effects of fiber similar to those in Fig 2 were observed in the cecum. Inulin increased the total cecal weight compared to the Control in our previous study [10]. In this study, Chow-feeding also led to a similar increase in the total cecal weight (0.39±0.06 g). Inulin- and Chow-fed mice had more microbial DNA per wet weight of cecal content compared to the Control-fed mice (93.7, 80.0 vs. 4.66 μg DNA/g wet weight). Dietary fiber did not affect the water content in the cecum with around 77% water per wet weight of cecal content in all three groups.

**Fig 3:**
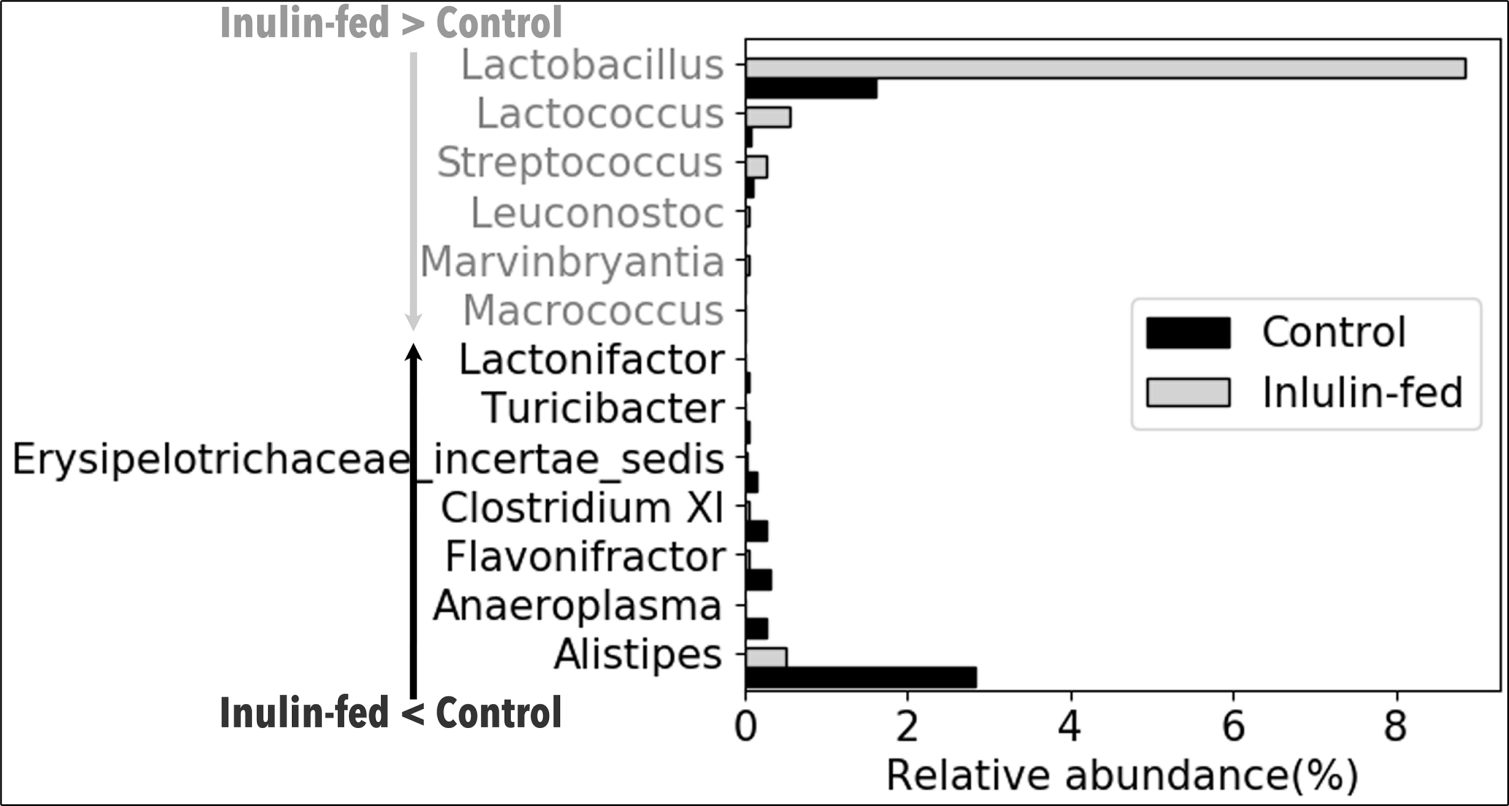
The effect of 8-wk AIN-93G diet containing purified fermentable fiber, inulin, on the relative abundance of fecal bacterial genera. Feces collected at two time points from the same group of mice (N = 7) were compared. Control feces were collected before the start of Inulin diet. Inulin-fed feces were collected after 8-wk Inulin-supplemented diet. The genera shown in the figure represent those that had significant difference (corrected p<0.05) between the two collections of feces and are listed following the ranking in abundance. The corrected p-value for *Clostridium XI* is 1.05E-02. The genera in light gray font have higher prevalence after 8-wk Inulin-supplemented diet. The genera in dark font have lower prevalence after 8-wk Inulin-supplemented diet.

**Fig 4:**
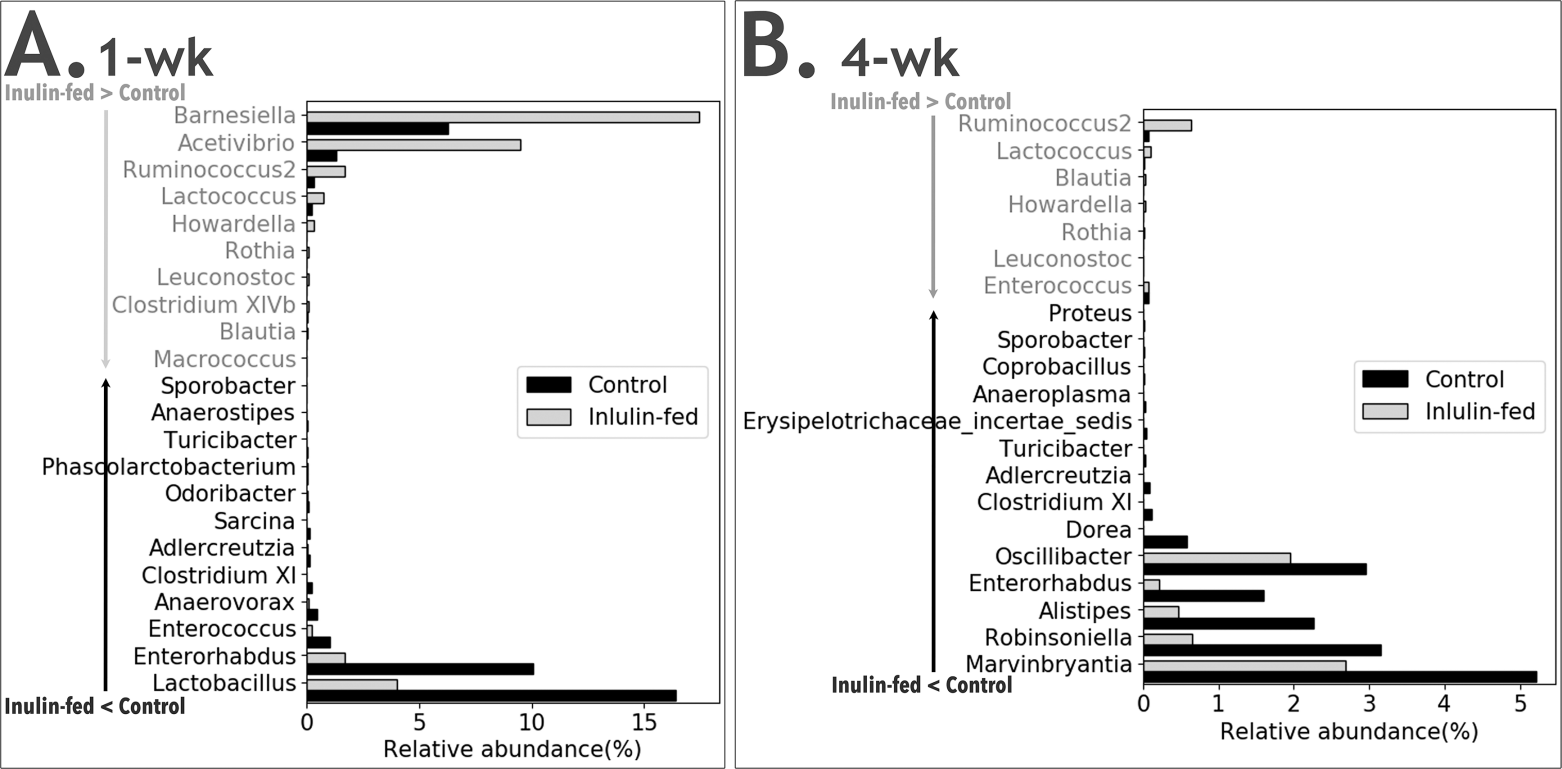
Comparison between mice consuming AIN-93G diet with or without purified fermentable fiber, inulin, on the relative abundance of fecal bacterial genera. Comparisons were made between the feces from two groups of age-matched mice after 1-wk of Control or Inulin (A); and between the feces from two groups of age-matched mice after 4-wk of Control or Inulin (B). N = 7–8 mice in each group. The genera shown in the figure represent those that had significant difference (corrected p<0.05) between the two groups of mice and are listed following the ranking in abundance. The corrected p-value for *Clostridium XI* is 1.71E-05 (A) and 4.05E-16 (B). The genera in light gray font have higher prevalence in the Inulin group. The genera in dark font have lower prevalence in the Inulin group.

**Fig 5:**
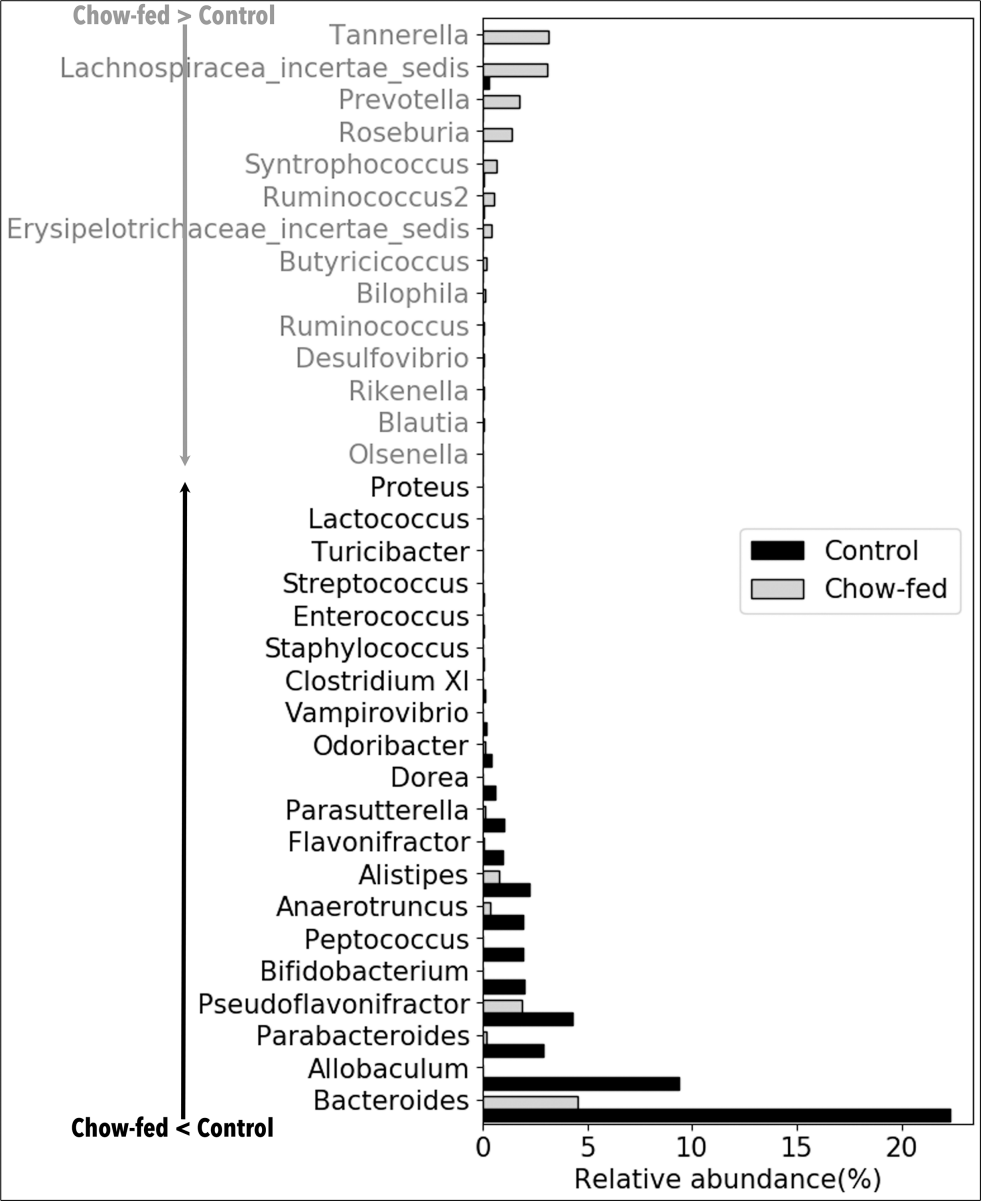
Comparison between mice consuming AIN-93G control diet and mice consuming chow with unpurified fibers on the relative abundance of fecal bacterial genera. Comparisons were made between the feces from mice after 4-wk of Control or chow. N = 5–7 mice in each group. The genera shown in the figure represent those that had significant difference (corrected p<0.05) between the two groups of mice and are listed following the ranking in abundance. The corrected p-value for *Clostridium XI* is 1.54E-10. The genera in light gray font have higher prevalence in the Chow group. The genera in dark font have lower prevalence in the Chow group.

**Fig 6:**
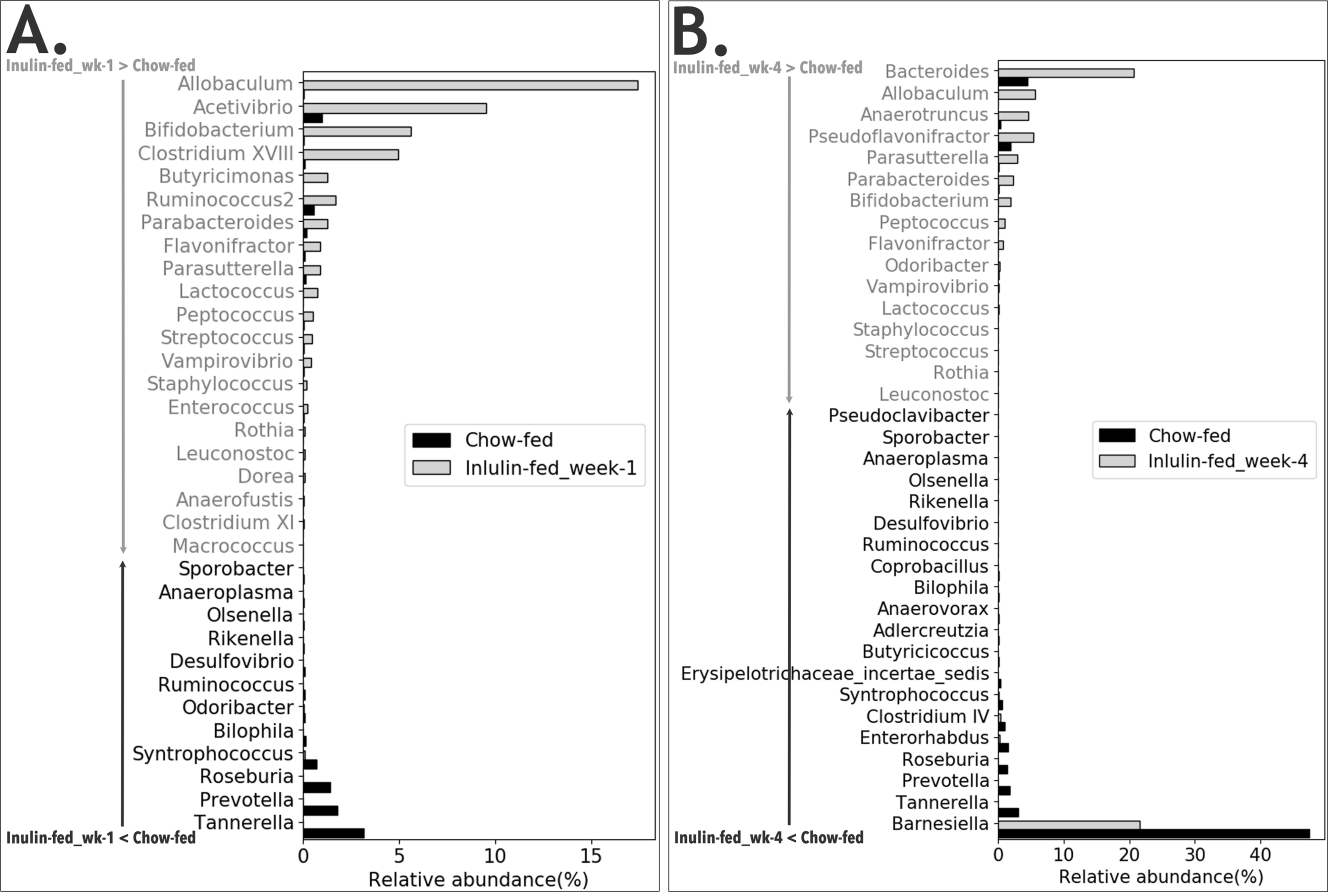
Comparison between mice consuming inulin-containing AIN-93G diet and mice consuming chow with unpurified fibers on the relative abundance of fecal bacterial genera. (A) Comparison between Chow-fed mice and mice after 1-wk Inulin feeding; (B) Comparison between Chow-fed mice and mice after 4-wk Inulin feeding. N = 5–8 mice in each group. The genera shown in the figure represent those that had significant difference (corrected p<0.05) between the two groups of mice and are listed following the ranking in abundance. The corrected p-value for *Clostridium XI* is 1.39E-2 and 0.992, respectively, for (A) and (B). The genera in light gray font have higher prevalence in the Inulin group. The genera in dark font have lower prevalence in the Inulin group.

The effect of fiber on the relative abundance of various bacterial genera was determined by comparing the microbiota from different dietary groups at different time points as shown in **Figs 3–6**. In Fig 3, the comparison was made between feces from the same group of mice before the start of Inulin and after 8-wk Inulin. Eight-wk Inulin-feeding led to differences in the fecal microbiota with the highest increase in the prevalence of *Lactobacillus*, a genus in the phylum Firmicutes with some probiotic species. The prevalence of *Alistipes*, a genus in the phylum Bacteroidetes, became lower upon the Inulin feeding. These two groups of samples were amplified using Illumina V1-V3 primer pair, which did not amplify *Bifidobacterium*. Nevertheless, qPCR did not detect any dietary effect on *Bifidobacterium*, a genus in the phylum Actinobacteria also with some probiotic species. In Fig 4A and 4B, feces from two groups of age-matched mice were compared. One-week and 4-wk Inulin feeding both affected the fecal microbiota but the effects were not the same. While both comparisons (Fig 4A and 4B) found no difference in the relative abundance of *Bifidobacterium*, *Lactobacillus* was lower in the Inulin group at the 1-wk comparison. Although no one genus was consistently increased by soluble fiber inulin in Figs 3 and 4, *Clostridium XI* was consistently decreased by Inulin-feeding.

Fig 5 shows a long list of genera that had significant difference between the intestinal microbiota of Chow-fed mice and Control mice. The prevalence of *Bifidobacterium* was lower in Chow-fed mice but *Lactobacillus* was not different between two groups. Similar to Figs 3 and 4, Chow-feeding with its multiple fiber types also decreased *Clostridium XI* in Fig 5. In Fig 6, fecal microbiota from the Inulin-fed mice was compared to that of the Chow-fed mice. At 1-wk after starting Inulin diet, the prevalence of *Clostridium XI* was higher than that in the Chow group (Fig 6A). However, this difference disappeared at 4-wk after Inulin diet (Fig 6B). *Bifidobacterium* was higher in the Inulin-fed groups at both time points (Fig 6).

The % relative abundance of *Clostridium XI* from all fecal samples of Control and Inulin groups that had been sequenced was summarized in **Table 1**. Table 1 includes fecal samples that were not used for the microbiota comparison because of the small group size.

**Table 1:** Summary of the prevalence of fecal *Clostridium XI* in mice given AIN93-G diet (Control diet) or AIN93-G diet supplemented with inulin (Inulin diet).

	Prevalence of *Clostridium XI* (% of total)^a^		
Duration of dietary treatment	Control diet	Inulin diet	P value
1 wk	0.191±0.109 (7)	0.010±0.017 (8)	4.2E-04
2 wk	0.251±0.238 (7)	0.003±0.006 (8)	0.011
4 wk	0.098±0.079 (7)	0±0 (8)	0.004
5 wk	0.079±0.020 (2)	0.001±0.001 (6)	2.5E-05
6 wk	0.375±0.147 (3)	0±0 (6)	2.7E-04
7 wk	0.423±0.175 (4)	0±0 (8)	2.8E-05
8 wk	0.301±0.076 (3)	0±0 (7)	3.0E-05
**Switch to Control diet after 8-wk Inulin diet**^b^			
1 wk	0±0 (4)		
2 wk	0±0 (3)		
3 wk	0±0 (5)		
4 wk	0.011±0.023 (4)^c^		

^**a**^Results were pooled from 2 runs of sequencing. Numbers in the parenthesis after the mean ± SD represent the group size. Student’s t-test was used to compare between mice of the same age under two different diets.

^b^After 8-wk Inulin diet, some mice from the Inulin diet group were switched to Control diet for another 4 weeks.

^c^At 4 weeks after the diet switching, fecal sample from one mouse showed a prevalence of *Clostridium XI* at 0.045% while the other three remains undetectable.

Before the start of the Inulin diet, mouse fecal samples of the Inulin diet group had a relative abundance of *Clostridium XI* at 0.197±±0.286%. As shown in Table 1, up to 8-wk of control diet did not appear to affect the prevalence of *Clostridium XI*. The same nutritionally balanced Inulin diet, in contrast, significantly reduced the presence of *Clostridium XI*. After only 4-wk of Inulin feeding, *Clostridium XI* became undetectable and remained very low or undetectable. Fecal samples from Chow-fed mice also had no detectable *Clostridium XI*. *Clostridium XI* was detected in cecal samples of Control diet-fed mice. After 8-wk Control diet, the cecal samples had a prevalence of *Clostridium XI* at 0.009±0.004%. In contrast, after 8-wk Inulin diet, the cecal samples had no detectable *Clostridium XI*. After 8-wk Inulin diet, some mice were switched to the Control diet. Fecal samples had no detectable *Clostridium XI* during the first 3 weeks after switching. At 4-wk after switching to the Control diet, the cecal samples still had no detectable *Clostridium XI* but one mouse (out of 4) had *Clostridium XI* DNA found in the fecal sample again. To validate our PCR amplification and sequencing procedures, technical duplicates were included. The relative abundance of *Clostridium XI* in the technical duplicates is summarized in **S1 Table**.

The microbiota data that were used in comparing the prevalence of all genera shown in Figs 3–6 were also used to analyze major bacterial genera and the results are shown in **S3–S6 Figs.** Except in S3 Fig where the V1-V3-based analysis could not detect *Bifidobacterium*, all V3-V4-based analysis found *Bifidobacterium* as a major genus (S4–S6 Figs). The genera that were consistently found to be major in **S3–S6 Figs** were *Allobaculum*, *Bacteroides*, *Clostridium XIVa*, and *Lactobacillus*. *Clostridium XI* was not in any list of major genera.

**Fig 7:**
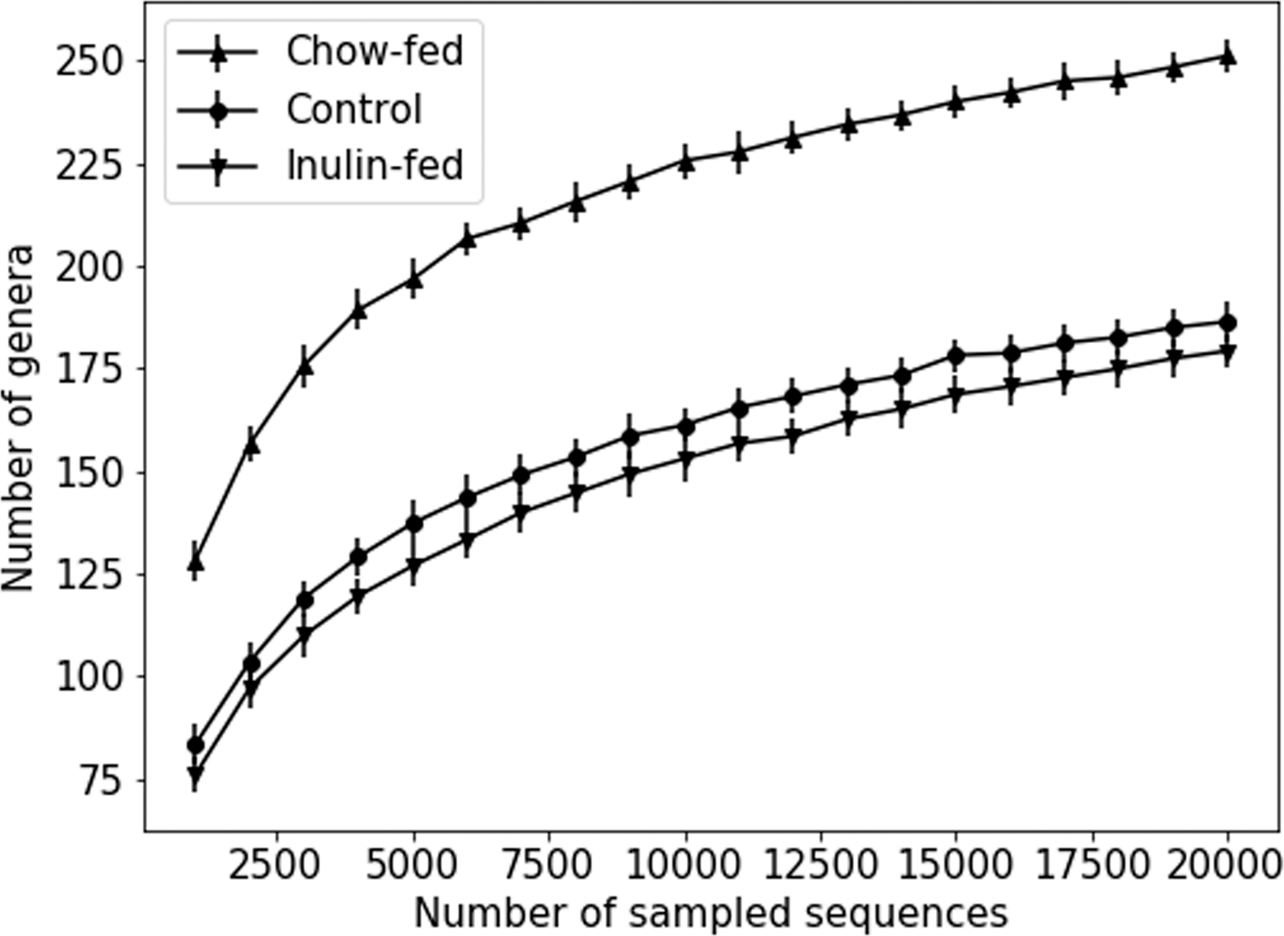
The effect of fiber on the fecal bacterial diversity. Fecal samples of the Control and Inulin groups were from 4-wk of respective dietary treatment. The data shown are means ± SEM with N = 5–8 mice in each group.

Higher bacterial diversity may be essential for the beneficial effect of fiber but human trials and animal studies did not observed a significant increase in diversity after the supplementation with purified soluble fibers [12, 13]. The fecal microbiota diversity shown as the number of genera was compared among mice consuming Control, Inulin and Chow and the results are shown in **Fig 7**. The number of genera was found to be consistently much higher in the fecal samples of Chow-fed mice compared to the Control mice independent of the number of sampled sequences. In contrast, Inulin feeding failed to increase the number of genera despite its similar ability to increase fecal bacterial load, density and reduce the presence of *Clostridium XI*.

## Discussion

Fiber intake was found to rapidly change human fecal microbiota [38]. Results of this mouse fiber-feeding study showed that 1-wk of inulin supplementation were sufficient to decrease the fecal presence of *Clostridium XI*. Persistent consumption of soluble fermentable fiber inulin is needed to maintain *Clostridium XI* at an undetectable level. Diet with only insoluble fiber cellulose failed to suppress *Clostridium XI*. Although cecal samples had overall lower prevalence of *Clostridium XI*, cecal samples also showed an absence of *Clostridium XI* after Inulin diet but not after Control diets. Unpurified soluble fibers as part of plant-based diet appear to have the same effect as inulin in controlling *Clostridium XI*. Because of the regular turnover of intestinal mucosa, fecal microbiota would include both luminal and colonized microbes. Future studies are needed to confirm that this observation can be translated to the reduction of *C*. *difficile* presence and its colonization at the species level in humans.

Our mouse observation is consistent with that of fiber-feeding studies done on other mammalian species. Decreased *Clostridium XI* was observed when dogs were supplemented with soybean husk [39], and when female rats fed high-fat/sucrose diet with inulin supplementation [40]. In an inulin-feeding study using transgenic mice as a model for inflammatory bowel disease [41], a decrease in intestinal inflammation was associated with higher total fecal bacteria and lower fecal and cecal *Clostridium XI* as well as lower fecal *C*. *difficile* toxin B. Although our study did not establish a dose-response curve, this reduction in *Clostridium XI* by soluble fiber could have important public health implication since only 10% of US adults met the fiber intake requirement based on a recent analysis of data from the National Health and Nutrition Examination Survey [42].

Higher soluble fiber to insoluble fiber ratio in the diet was linked to more intestinal fermentation by-products in dogs [15]. The results of their indirect measurement of bacterial load are consistent with the results of our direct fecal bacteria quantification. None of the previous studies examined fecal microbiota and 24-hr fecal bacterial load/density simultaneously [12]. Compared to the soluble fiber-free AIN93-G diet, Inulin diet and Chow increased total fecal bacteria load and bacterial density. The higher total bacteria population likely contributes to the lower presence or even absence of *Clostridium XI* by soluble fiber intake. Most previous studies emphasize the importance of particular microbiota composition or diversity but in this study, Chow and Inulin diet affected the microbiota composition differently and only Chow-feeding increased diversity. Our observation on the possible importance of fecal bacteria load and bacterial density is consistent with indirect clinical evidences. Successful fecal microbiome transplantation to treat *C*. *difficile* infection did not depend on the presence of particular genera [43] and did not always lead to changes in diversity [44]. Antibiotic usage and cancer chemotherapy are known to reduce total commensal bacteria and have been repeatedly found as risk factors for *C*. *difficile* infection [1, 45–47]. Antibiotics designed to preserve commensal bacteria showed more effectiveness when used for the control of *C*. *difficile* infection [48].

Chow-feeding but not insulin supplementation promoted diversity likely because it contains more varieties of fiber (compared to the purified AIN-93G diet). Sugar beet pulp supplementation of purified diet in pigs was also found to increase fecal bacterial diversity [49]. In contrast, inulin addition to a modified high fat AIN-93G diet [50], or to a sugar beet pulp diet [49] did not lead to more diversity. The addition of functional fiber other than inulin also did not increase diversity [51, 52]. These observations on microbiome diversity further support a major role of fiber from the host diet as an important, if not primary, driving force for bacteria population dynamics [38].

Inulin intake was found to promote the conversion of primary bile salt to the secondary bile salts [10]. A decrease in the ratio of primary to secondary bile salt in the large intestine may be one mechanism contributing to the suppression of *Clostridium XI* by soluble fiber intake. A higher ratio of primary to secondary bile salts was found to promote the colony formation of *C*. *difficile* [53, 54]. Antibiotic treatment that favored the growth of *C*. *difficile*, on the other hand, decreases the conversion of the primary bile salts to the secondary bile salts in the intestine [54]. Also, fecal microbiome transplantation that cured *C*. *difficile* infection was found to restore normal fecal bile salt composition [55].

Fiber fermentation by microbiota can lead to the production of unique metabolites including short-chain fatty acids. The potential anti-inflammatory effects of these metabolites have been reviewed [12]. However, none of the studies that examined the direct role of short-chain fatty acids and other metabolites included the quantification of *C*. *difficile* or *Clostridium XI*. Also, decreases in fecal *Clostridium XI* and *C*. *difficile* toxin B were not always associated with higher fecal short-chain fatty acids [41].

Other mechanisms that can contribute to the colonization resistance have also been proposed but a direct testing of these microbe-microbe interaction or host-commensal interaction is challenging. For example, the growth of *C*. *difficile* may be modulated through bacterial quorum-sensing signals [56, 57]. Under stress conditions, changes in the intestinal gene expression were reported in inulin-supplemented animals [11, 58]. These gene expression changes may provide colonization resistance.

Developing infant gut microbiota has unique features compared to that of adults [59, 60]. In our mice study, newly weaned mice also showed a distinct pattern of microbiota (comparing S3 Fig left panel with S4–S6 Figs). However, the unique infant microbiota features may not be a result of young age. Neither the human infant observations nor our mouse study directly test the effect of age independent of dietary differences, milk in infants vs. table/solid food upon weaning. Instead, an importance of diet in shaping intestinal microbiota has been reviewed [12].

Mice under Control and Inulin diet ate less compared to Chow-fed mice. However, the total energy intake was similar among three groups, which led to a similar weight gain. This is consistent with our previous observation [10]. The results of our studies do not support any short-term or long-term role of fiber in the energy balance under moderate fat intake. There was also no apparent association between microbiome composition/diversity and body weight. This conclusion is consistent with the proposed loose coupling between energy intake and energy expenditure on a daily basis [61]. The observed similar weight gain between mice fed purified inulin diet and mice given chow rich in dietary fiber also did not support any structure-dependent activities of fiber in energy balance.

## Conclusions

Purified and unpurified fiber, without affecting body weight, can both increase the fecal bacterial load/density and reduce the prevalence of *Clostridium XI*. Higher prevalence of particular probiotic bacterial genera or higher microbial diversity is not necessary. Future studies on the analysis of intestinal microbiota and on health implications should take the 24-hr fecal bacterial load and density into consideration. A recent human study also recommended quantitative microbiota profiling taking the bacterial density into the consideration [62].

## Supporting information

S1 Table *Clostridium XI* prevalence determined from the technical duplicates of cecal and fecal DNA samples. (DOCX)

S1 Fig qPCR analysis of Bifidobacterium. (TIF)

S2 Fig The significant correlation between daily food intake, daily neutral detergent fiber intake, and daily fecal output in chow-fed mice. (TIF)

S3 Fig The comparison between major fecal bacteria genera of mice before (Control) and after 8-wk feeding of inulin -containing diet (Inulin). (TIF)

S4 Fig The comparison between major fecal bacteria genera of age-matched mice groups after (A) 1-wk or (B) 4-wk feeding of Control or inulin-containing diet.
(TIF)

S5 Fig The comparison between major fecal bacteria genera of adult mice groups fed Control diet or wheat, corn and soybean-based rodent chow (Chow). (TIF)

S6 Fig The comparison between major fecal bacteria genera of mice groups fed wheat, corn and soybean-based rodent chow (Chow) and mice at (A) 1 week or (B) 4 weeks after the start of inulin-containing diet.
(TIF)

## Abbreviations

Control: AIN-93G diet
Chow: wheat, corn and soybean-based commercial rodent chow
Inulin: AIN-93G diet supplemented with 5% (wt:wt) inulin
C. difficile: *Clostridium difficile*
OTU: operational taxonomic units

## References

[1] Razik R, Rumman A, Bahreini Z, McGeer A, Nguyen G. Recurrence of Clostridium difficile infection in patients with Inflammatory Bowel Disease: The RECIDIVISM Study. Am J Gastroenterol. 2016;111(8):1141–6. 10.1038/ajg.2016.187 27215924

[2] Ma G, Brensinger C, Wu Q, Lewis J. Increasing Incidence of Multiply Recurrent Clostridium difficile Infection in the United States: A Cohort Study. Ann Intern Med. 2017;167(3):152–8. 10.7326/M16-2733 28672282

[3] Lopetuso L, Scaldaferri F, Petito V, Gasbarrini A. Commensal Clostridia: leading players in the maintenance of gut homeostasis. Gut Pathog. 2013;5(1):23 10.1186/1757-4749-5-23 23941657 PMC3751348

[4] McClane BA, Lyerly DM, Wilkins TD. Enterotoxic Clostridia: Clostridium perfringenes Type A and Clostridium difficile In: Fischetti VA, Novick RP, Ferretti JJ, Portnoy DA, Rood JI, editors. Gram-positive Pathogens. 2nd ed Washington, D.C: ASM Press; 2006 p. 703–14.

[5] Zhang L, Dong D, Jiang C, Li Z, Wang X, Peng Y. Insight into alteration of gut microbiota in Clostridium difficile infection and asymptomatic C. difficile colonization. Anaerobe. 2015;34:1–7. 10.1016/j.anaerobe.2015.03.008 25817005

[6] Adlerberth I, Huang H, Lindberg E, Åberg N, Hesselmar B, Saalman R, et al Toxin-producing Clostridium difficile strains as long-term gut colonizers in healthy infants. J Clin Microbiol. 2014;52(1):173–9. 10.1128/JCM.01701-13 24172156 PMC3911410

[7] Reeves PG, Nielsen FH, Fahey GCJ. AIN-93 purified diets for laboratory rodents: final report of the American Institute of Nutrition Ad Hoc Writing Committee on the reformulation of the AIN-76A rodent diet. J Nutr. 1993;123:1939–51. 10.1093/jn/123.11.1939 8229312

[8] Niness K. Inulin and oligofructose: what are they? J Nutr. 1999;129:1402S–6S. 10.1093/jn/129.7.1402S 10395607

[9] Roberfroid M. Introducing inulin-type fructans. Br J Nutr. 2005;93 Suppl 1:S13–25. 15877886 10.1079/bjn20041350

[10] Kuo S-M, Merhige PM, Hagey LR. The effect of dietary prebiotics and probiotics on body weight, large intestine indices, and fecal bile acid profile in wildtype and IL10-/- mice. PlosOne. 2013;8(3):e60270. 10.1371/journal.pone.0060270 PMC3605333 23555939

[11] Kuo S-M, Chan W-C, Hu Z. Wild-type and IL10-null mice have differential colonic epithelial gene expression responses to dietary supplementation with synbiotic Bifidobacterium animalis Subspecies lactis and inulin. J Nutr. 2014;144:245–51. 10.3945/jn.113.185249 24381223

[12] Kuo S-M. Does modification of the large intestinal microbiome contribute to the anti-inflammatory activity of fermentable fiber? Curr Dev Nutr. 2018;2:nzx004. 10.3945/cdn.117.001180 PMC6201682 30377676

[13] So D, Whelan K, Rossi M, Morrison M, Holtmann G, Kelly J, et al Dietary fiber intervention on gut microbiota composition in healthy adults: a systematic review and meta-analysis. Am J Clin Nutr. 2018;107:1–19. 10.1093/ajcn/nqx066 29757343

[14] Navarro D, Bruininx E, de Jong L, Stein H. Effects of physicochemical characteristics of feed ingredients on the apparent total tract digestibility of energy, dry matter and nutrients by growing pigs. J Anim Sci. 2018;(Epub ahead of print]. 10.1093/jas/sky149 PMC6095346 29688508

[15] Swanson K, Grieshop C, Clapper G, Shields RJ, Belay T, Merchen N, et al Fruit and vegetable fiber fermentation by gut microflora from canines. J Anim Sci. 2001;79:919–26. 11325198 10.2527/2001.794919x

[16] Surawicz C, Brandt L, Binion D, Ananthakrishnan A, Curry S, Gilligan P, et al Guidelines for diagnosis, treatment, and prevention of Clostridium difficile infections. Am J Gastroenterol. 2013;108(4):478–98. 10.1038/ajg.2013.4 23439232

[17] Pérez-Cobas A, Moya A, Gosalbes M, A. L. Colonization Resistance of the Gut Microbiota against Clostridium difficile. Antibiotics. 2015;4(3):337–57. 10.3390/antibiotics4030337 27025628 PMC4790290

[18] Carroll I, Ringel-Kulka T, Siddle J, Klaenhammer T, Ringel Y. Characterization of the fecal microbiota using high-throughput sequencing reveals a stable microbial community during storage. PLoS One. 2012;7:e46953 10.1371/journal.pone.0046953 23071673 PMC3465312

[19] Yu Y, Majumdar A, Nechvatal J, Ram J, Basson M, Heilbrun L, et al Exfoliated cells in stool: a source for reverse transcription-PCR-based analysis of biomarkers of gastrointestinal cancer. Cancer Epidemiol Biomarkers Prev. 2008;17(2):455–8. 10.1158/1055-9965.EPI-07-2515 18268130 PMC3860274

[20] Hsieh Y, Peterson C, Raggio A, Keenan M, Martin R, Ravussin E, et al Impact of different fecal processing methods on assessments of bacterial diversity in the human intestine. Front Microbiol. 2016;7:1643 10.3389/fmicb.2016.01643 27812352 PMC5071325

[21] Koch A. Turbidity measurements of bacterial cultures in some available commercial instruments. Anal Biochem. 1970;38(1):252–9. 4920662 10.1016/0003-2697(70)90174-0

[22] Myllyluoma E, Veijola L, Ahlroos T, Tynkkynen S, Kankuri E, Vapaatalo H, et al Probiotic supplementation improves tolerance to Helicobacter pylori eradication therapy—a placebo-controlled, double-blind randomized pilot study. Aliment Pharmacol Ther. 2005;21(10):1263–72. 10.1111/j.1365-2036.2005.02448.x 15882248

[23] Myllyluoma E, Kajander K, Mikkola H, Kyrönpalo S, Rasmussen M, Kankuri E, et al Probiotic intervention decreases serum gastrin-17 in Helicobacter pylori infection. Dig Liver Dis. 2007;39:516–23. 10.1016/j.dld.2007.02.015 17433799

[24] Kuo S-M, MacLean ME, McCormick K, Wilson JX. Gender and sodium-ascorbate transporter isoforms determine ascorbate concentrations in mice. J Nutr. 2004;134(9):2216–21. 10.1093/jn/134.9.2216 15333707

[25] Masso-Welch PA, Merhige PM, Veeranki OLM, Kuo S-M. Loss of IL-10 decreases mouse postpubertal mammary gland development in the absence of inflammation. Immunol Invest. 2012;41(5):521–37. 10.3109/08820139.2012.684193 22594921

[26] Zheng W, Tsompana M, Ruscitto A, Sharma A, Genco R, Sun Y, et al An accurate and efficient experimental approach for characterization of the complex oral microbiota. Microbiome. 2015;3:48 10.1186/s40168-015-0110-9 26437933 PMC4593206

[27] Cock P, Fields C, Goto N, Heuer M, Rice P. The Sanger FASTQ file format for sequences with quality scores, and the Solexa/Illumina FASTQ variants. Nucleic Acids Res. 2010;38(6):1767–71. 10.1093/nar/gkp1137 20015970 PMC2847217

[28] Zhang J, Kobert K, Flouri T, Stamatakis A. PEAR: a fast and accurate Illumina Paired-End reAd mergeR. Bioinformatics. 2014;30(5):614–20. 10.1093/bioinformatics/btt593 24142950 PMC3933873

[29] Ewing B, Hillier L, Wendl M, Green P. Base-calling of automated sequencer traces using phred. I. Accuracy assessment. Genome Res. 1998;8(3):175–85. 9521921 10.1101/gr.8.3.175

[30] Altschul S, Gish W, Miller W, Myers E, Lipman D. Basic local alignment search tool. J Mol Biol. 1990;215:403–10. 10.1016/S0022-2836(05)80360-2 2231712

[31] Cole J, Chai B, Farris R, Wang Q, Kulam S, McGarrell D, et al The Ribosomal Database Project (RDP-II): sequences and tools for high-throughput rRNA analysis. Nucleic Acids Res. 2005;33:294–6. 10.1093/nar/gki038 PMC539992 15608200

[32] Love M, Huber W, Anders S. Moderated estimation of fold change and dispersion for RNA-seq data with DESeq2. Genome Biol. 2014;15(12):550 10.1186/s13059-014-0550-8 25516281 PMC4302049

[33] Benjamini Y, Hochberg Y. Controlling the false discovery rate: a practical and powerful approach to multiple testing. JRSSB. 1995;57:289–300.

[34] Caporaso J, Kuczynski J, Stombaugh J, Bittinger K, Bushman F, Costello E, et al QIIME allows analysis of high-throughput community sequencing data. Nature Methods. 2010;7:335–6. 10.1038/nmeth.f.303 20383131 PMC3156573

[35] Mai V, Torrazza R, Ukhanova M, Wang X, Sun Y, Li N, et al Distortions in development of intestinal microbiota associated with late onset sepsis in preterm infants. PLoS One. 2013;8:e52876 10.1371/journal.pone.0052876 23341915 PMC3544792

[36] Penders J, Vink C, Driessen C, London N, Thijs C, Stobberingh E. Quantification of Bifidobacterium spp., Escherichia coli and Clostridium difficile in faecal samples of breast-fed and formula-fed infants by real-time PCR. FEMS Microbiol Lett. 2005;243(1):141–7. 10.1016/j.femsle.2004.11.052 15668012

[37] Kirk RE. Multiple comparison tests Experimental Design: Procedures for the Behavioral Sciences. 4th ed U.S.A: SAGE Publications, Inc 2013 p. 154–208.

[38] David L, Materna A, Friedman J, Campos-Baptista M, Blackburn M, Perrotta A, et al Host lifestyle affects human microbiota on daily timescales. Genome Biol. 2014;15(7):R89 10.1186/gb-2014-15-7-r89 25146375 PMC4405912

[39] Myint H, Iwahashi Y, Koike S, Kobayashi Y. Effect of soybean husk supplementation on the fecal fermentation metabolites and microbiota of dogs. Anim Sci J. 2017; [Epub ahead of print]. 10.1111/asj.12817 28568309

[40] Paul H, Bomhof M, Vogel H, Reimer R. Diet-induced changes in maternal gut microbiota and metabolomic profiles influence programming of offspring obesity risk in rats. Sci Rep. 2016;6:20683 10.1038/srep20683 26868870 PMC4751613

[41] Koleva P, Valcheva R, Sun X, Gänzle M, Dieleman L. Inulin and fructo-oligosaccharides have divergent effects on colitis and commensal microbiota in HLA-B27 transgenic rats. Br J Nutr. 2012;108(9):1633–43. 10.1017/S0007114511007203 22243836

[42] McGill C, Birkett A, Fulgonii Iii V. Healthy Eating Index-2010 and food groups consumed by US adults who meet or exceed fiber intake recommendations NHANES 2001–2010. Food Nutr Res. 2016;60:29977 10.3402/fnr.v60.29977 27098562 PMC4838991

[43] Borgia G, Maraolo A, Foggia M, Buonomo A, Gentile I. Fecal microbiota transplantation for Clostridium difficile infection: back to the future. Expert Opin Biol Ther. 2015;15(7):1001–14. 10.1517/14712598.2015.1045872 26063385

[44] Shahinas D, Silverman M, Sittler T, Chiu C, Kim P, Allen-Vercoe E, et al Toward an understanding of changes in diversity associated with fecal microbiome transplantation based on 16S rRNA gene deep sequencing. MBio. 2012;3:e00338–12. 10.1128/mBio.00338-12 23093385 PMC3482503

[45] Tschudin-Sutter S, Tamma P, Milstone A, Perl T. Predictors of first recurrence of Clostridium difficile infections in children. Pediatr Infect Dis J. 2014;33:414–6. 10.1097/INF.0000000000000108 24168983

[46] Dantes R, Mu Y, Hicks L, Cohen J, Bamberg W, Beldavs Z, et al Association between outpatient antibiotic prescribing practices and community-associated Clostridium difficile infection. Open Forum Infect Dis. 2015;2(3):ofv113 10.1093/ofid/ofv113 26509182 PMC4551478

[47] Peretz A, Shlomo I, Nitzan O, Bonavina L, Schaffer P, Schaffer M. Clostridium difficile Infection: Associations with Chemotherapy, Radiation Therapy, and Targeting Therapy Treatments. Curr Med Chem. 2016;23(39):4442–9. 27804875 10.2174/0929867323666161028162018

[48] Sullivan K, Spooner L. Fidaxomicin: a macrocyclic antibiotic for the management of Clostridium difficile infection. Ann Pharmacother. 2010;44(2):352–9. 10.1345/aph.1M351 20071495

[49] Konstantinov S, Zhu W, Williams B, Tamminga S, Vos W, Akkermans A. Effect of fermentable carbohydrates on piglet faecal bacterial communities as revealed by denaturing gradient gel electrophoresis analysis of 16S ribosomal DNA. FEMS Microbiol Ecol. 2003;43(2):225–35. 10.1111/j.1574-6941.2003.tb01062.x 19719683

[50] Liu T, Cephas K, Holscher H, Kerr K, Mangian H, Tappenden K, et al Nondigestible fructans alter gastrointestinal barrier function, gene expression, histomorphology, and the microbiota profiles of diet-Induced obese C57BL/6J mice. J Nutr. 2016;146(5):949–56. 10.3945/jn.115.227504 27052535

[51] Umu Ö, Frank J, Fangel J, Oostindjer M, da Silva C, Bolhuis E, et al Resistant starch diet induces change in the swine microbiome and a predominance of beneficial bacterial populations. Microbiome. 2015;3:16 10.1186/s40168-015-0078-5 25905018 PMC4405844

[52] Ghaffarzadegan T, Marungruang N, Fåk F, Nyman M. Molecular properties of guar gum and pectin modify cecal bile acids, microbiota, and plasma lipopolysaccharide-binding protein in rats. PLoS One. 2016;11(6):e0157427 10.1371/journal.pone.0157427 27315087 PMC4912110

[53] Sorg J, Sonenshein A. Inhibiting the initiation of Clostridium difficile spore germination using analogs of chenodeoxycholic acid, a bile acid. J Bacteriol. 2010;192(19):4983–90. 10.1128/JB.00610-10 20675492 PMC2944524

[54] Giel J, Sorg J, Sonenshein A, Zhu J. Metabolism of bile salts in mice influences spore germination in Clostridium difficile. PLoS One. 2010;5:e8740 10.1371/journal.pone.0008740 20090901 PMC2806926

[55] Weingarden A, Chen C, Bobr A, Yao D, Lu Y, Nelson V, et al Microbiota transplantation restores normal fecal bile acid composition in recurrent Clostridium difficile infection. Am J Physiol Gastrointest Liver Physiol. 2014;306(4):G310–9. 10.1152/ajpgi.00282.2013 24284963 PMC3920123

[56] Hopkins M, Macfarlane G. Nondigestible oligosaccharides enhance bacterial colonization resistance against Clostridium difficile in vitro. Appl Environ Microbiol. 2003;69(4):1920–7. 10.1128/AEM.69.4.1920-1927.2003 12676665 PMC154806

[57] Ðapa T, Leuzzi R, Ng Y, Baban S, Adamo R, Kuehne S, et al Multiple factors modulate biofilm formation by the anaerobic pathogen Clostridium difficile. J Bacteriol. 2013;195(3):545–55. 10.1128/JB.01980-12 23175653 PMC3554014

[58] Matsumoto K, Ichimura M, Tsuneyama K, Moritoki Y, Tsunashima H, Omagari K, et al Fructo-oligosaccharides and intestinal barrier function in a methionine-choline-deficient mouse model of nonalcoholic steatohepatitis. PLoS One. 2017;12(6):e0175406 10.1371/journal.pone.0175406 28632732 PMC5478096

[59] Palmer C, Bik E, DiGiulio D, Relman D, Brown P. Development of the human infant intestinal microbiota. PLoS Biol. 2007;5:e177 10.1371/journal.pbio.0050177 17594176 PMC1896187

[60] Koenig J, Spor A, Scalfone N, Fricker A, Stombaugh J, Knight R, et al Succession of microbial consortia in the developing infant gut microbiome. Proc Natl Acad Sci U S A. 2011;108 Suppl 1:4578–85. 20668239 10.1073/pnas.1000081107 PMC3063592

[61] Drenowatz C. Reciprocal Compensation to Changes in Dietary Intake and Energy Expenditure within the Concept of Energy Balance. Adv Nutr. 2015;6(5):592–9. 10.3945/an.115.008615 26374181 PMC4561833

[62] Vandeputte D, Kathagen G, D'hoe K, Vieira-Silva S, Valles-Colomer M, Sabino J, et al Quantitative microbiome profiling links gut community variation to microbial load. Nature. 2017;551(7681):507–11. 10.1038/nature24460 29143816

