# Supplementary material for: Dietary or supplemental fermentable fiber intake reduces the presence of Clostridium XI in mouse intestinal microbiota: The importance of higher fecal bacterial load and density
Source: PLoS One. 2018 Oct 2;13(10):e0205055. doi: 10.1371/journal.pone.0205055 (PMC6168175; doi:10.1371/journal.pone.0205055)
Supplement: S1 Table — (DOCX) [file pone.0205055.s001.docx]

**S1 Table**

***Clostridium XI* prevalence determined from the technical duplicates of cecal and fecal DNA samples.**

|  | **Prevalence of *Clostridium XI* (% of total)^a^** | |
| --- | --- | --- |
| **Sample type** | Duplicate 1 | Duplicate 2 |
| Cecum DNA1 | 0.007 | 0.014 |
| Cecum DNA2 | 0 | 0 |
| Fecal DNA1 | 0.038 | 0.027 |
| Fecal DNA2 | 0.262 | 0.217 |

^a^Duplicate DNA samples were amplified, sequenced and analyzed independently.
