# Supplementary figures and images for: Dietary or supplemental fermentable fiber intake reduces the presence of Clostridium XI in mouse intestinal microbiota: The importance of higher fecal bacterial load and density
Source: PLoS One. 2018 Oct 2;13(10):e0205055. doi: 10.1371/journal.pone.0205055 (PMC6168175; doi:10.1371/journal.pone.0205055)

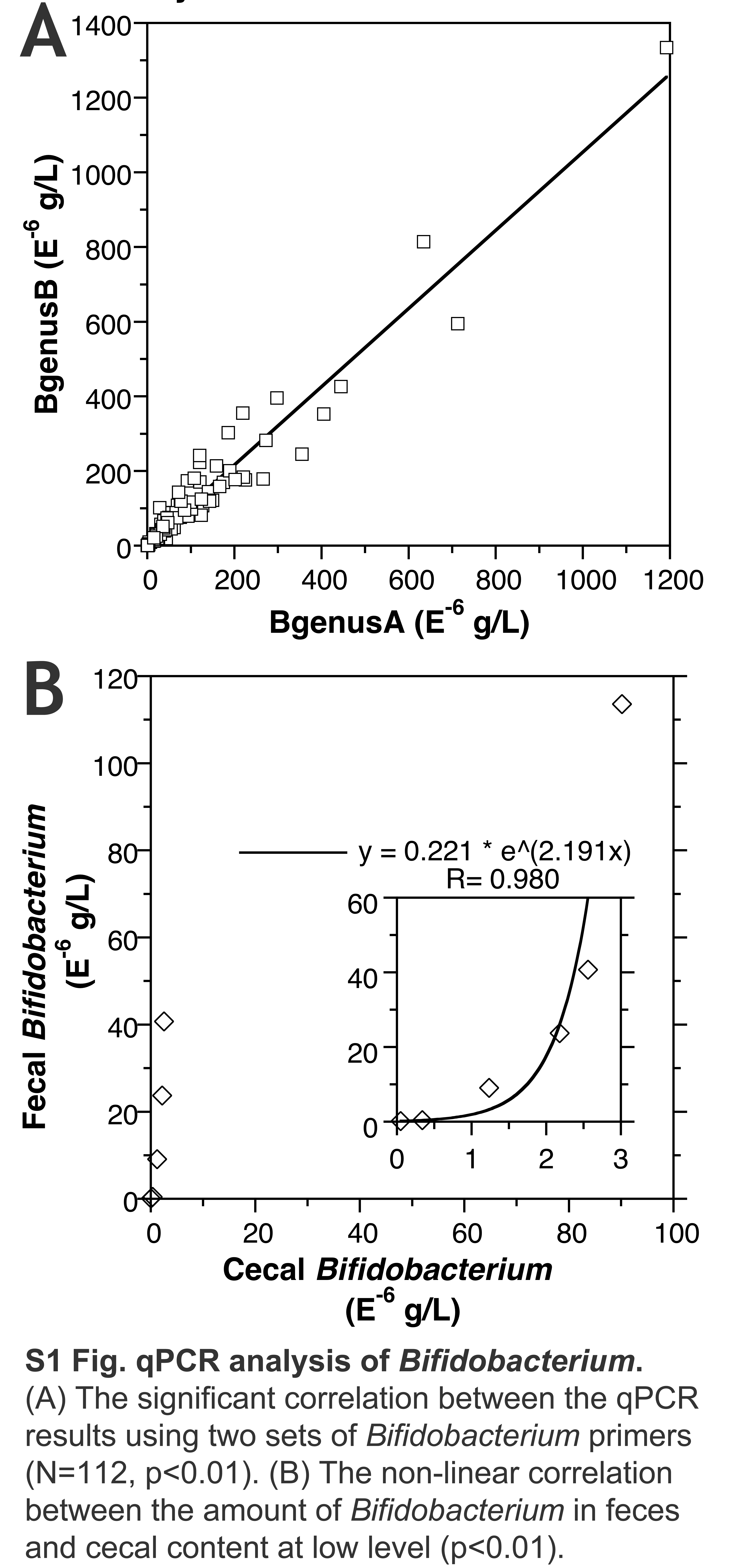

Supplement: S1 Fig — (TIF) [file pone.0205055.s002.tif]

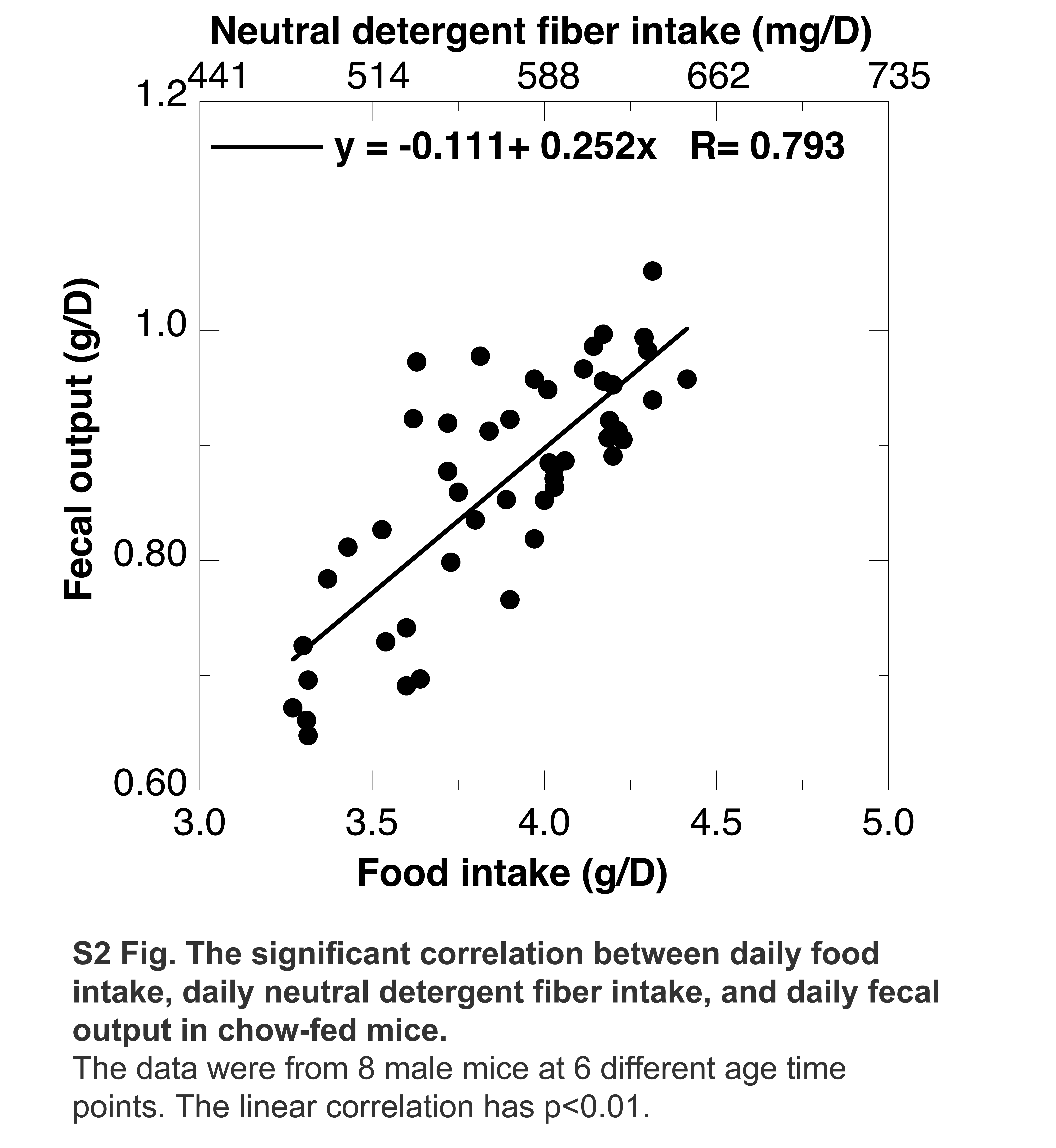

Supplement: S2 Fig — (TIF) [file pone.0205055.s003.tif]

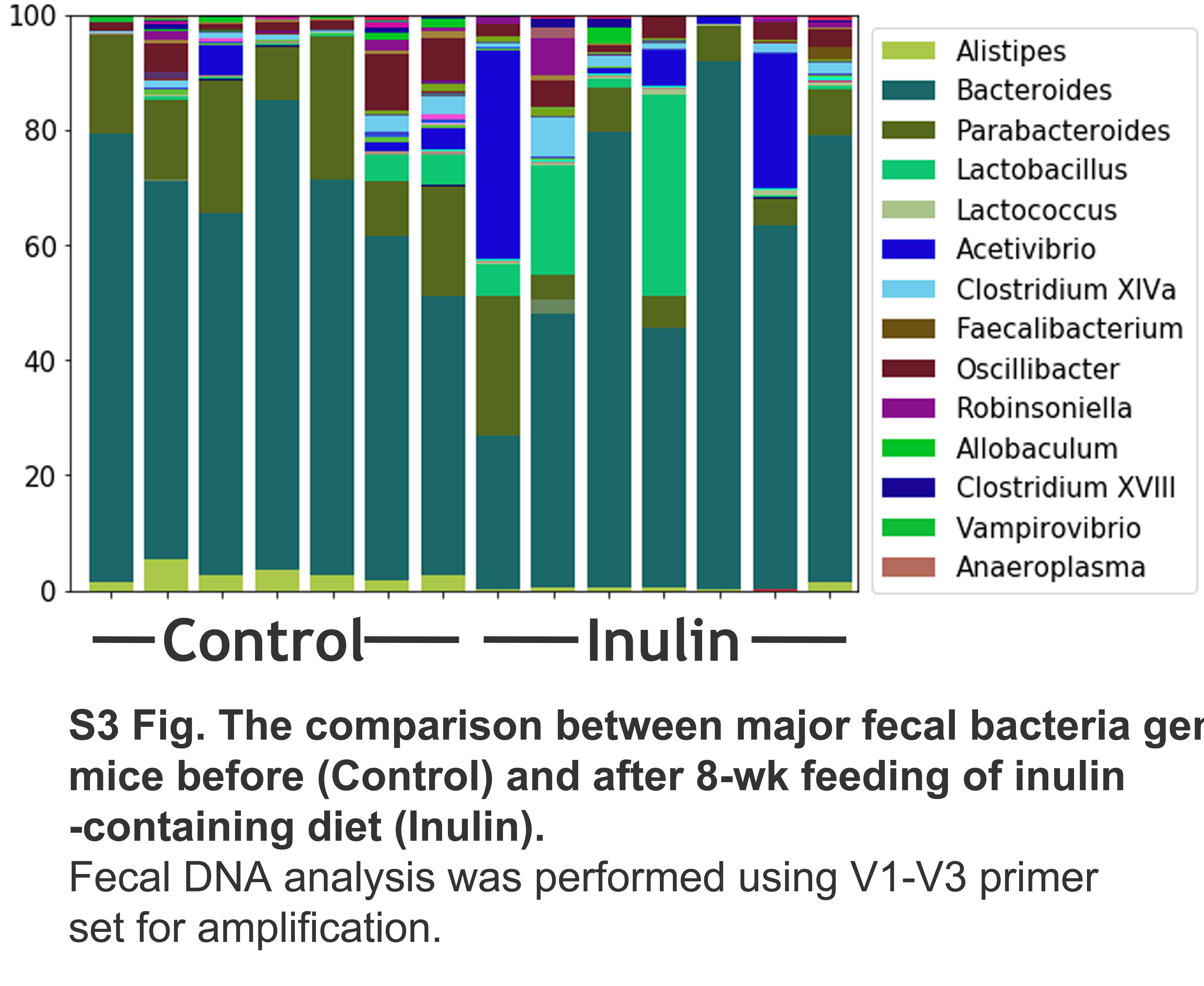

Supplement: S3 Fig — (TIF) [file pone.0205055.s004.tif]

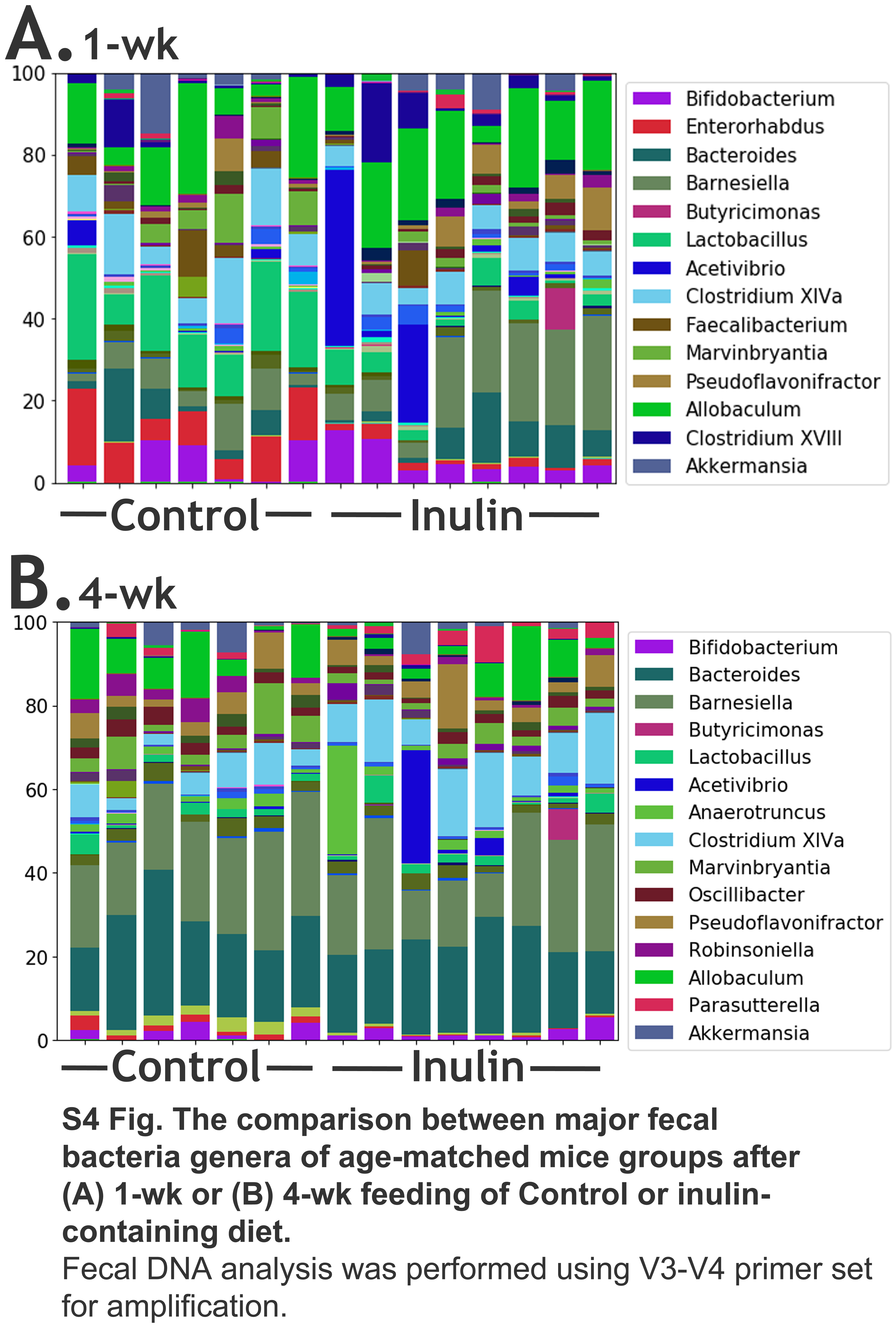

Supplement: S4 Fig — The comparison between major fecal bacteria genera of age-matched mice groups after (A) 1-wk or (B) 4-wk feeding of Control or inulin-containing diet. (TIF) [file pone.0205055.s005.tif]

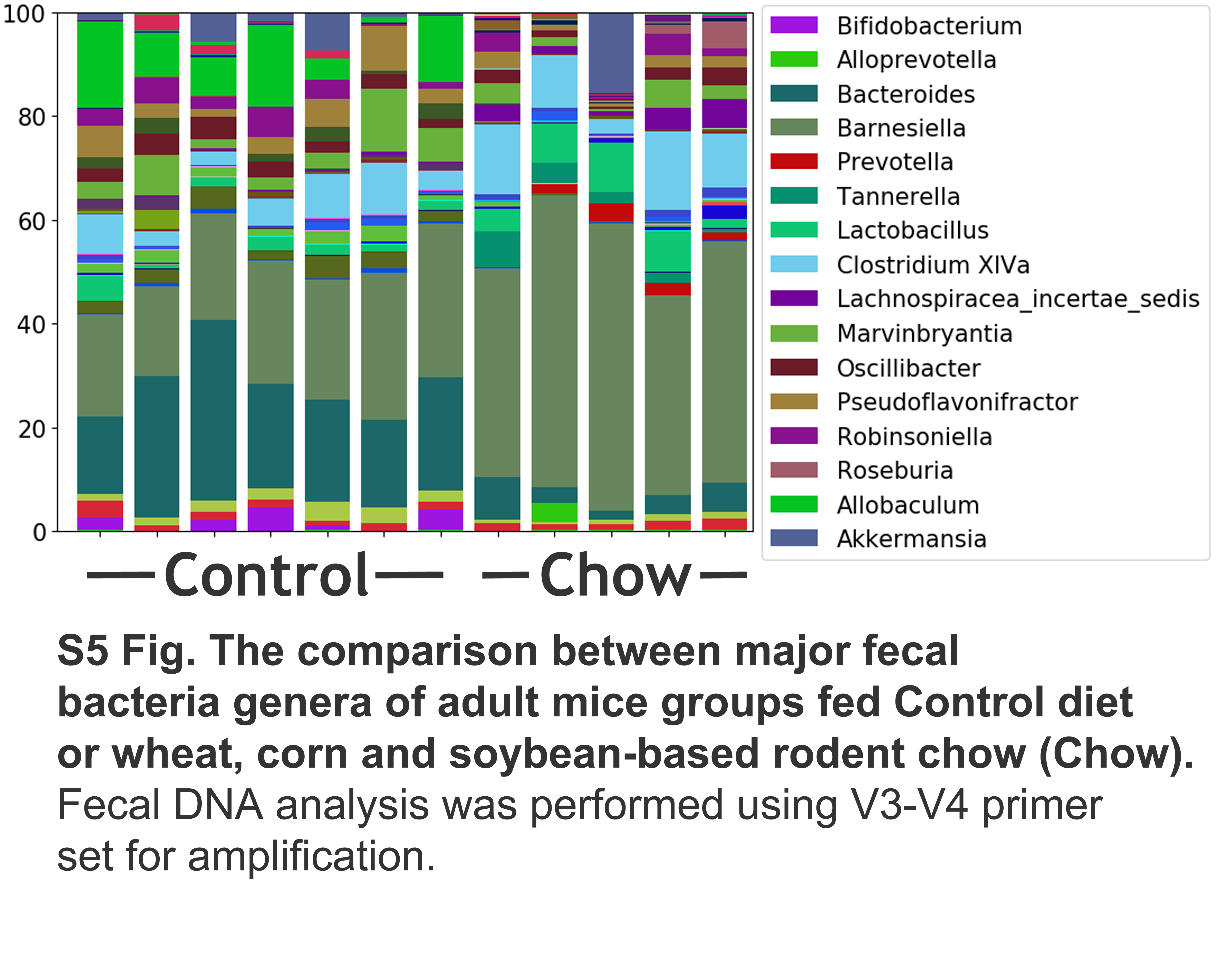

Supplement: S5 Fig — (TIF) [file pone.0205055.s006.tif]

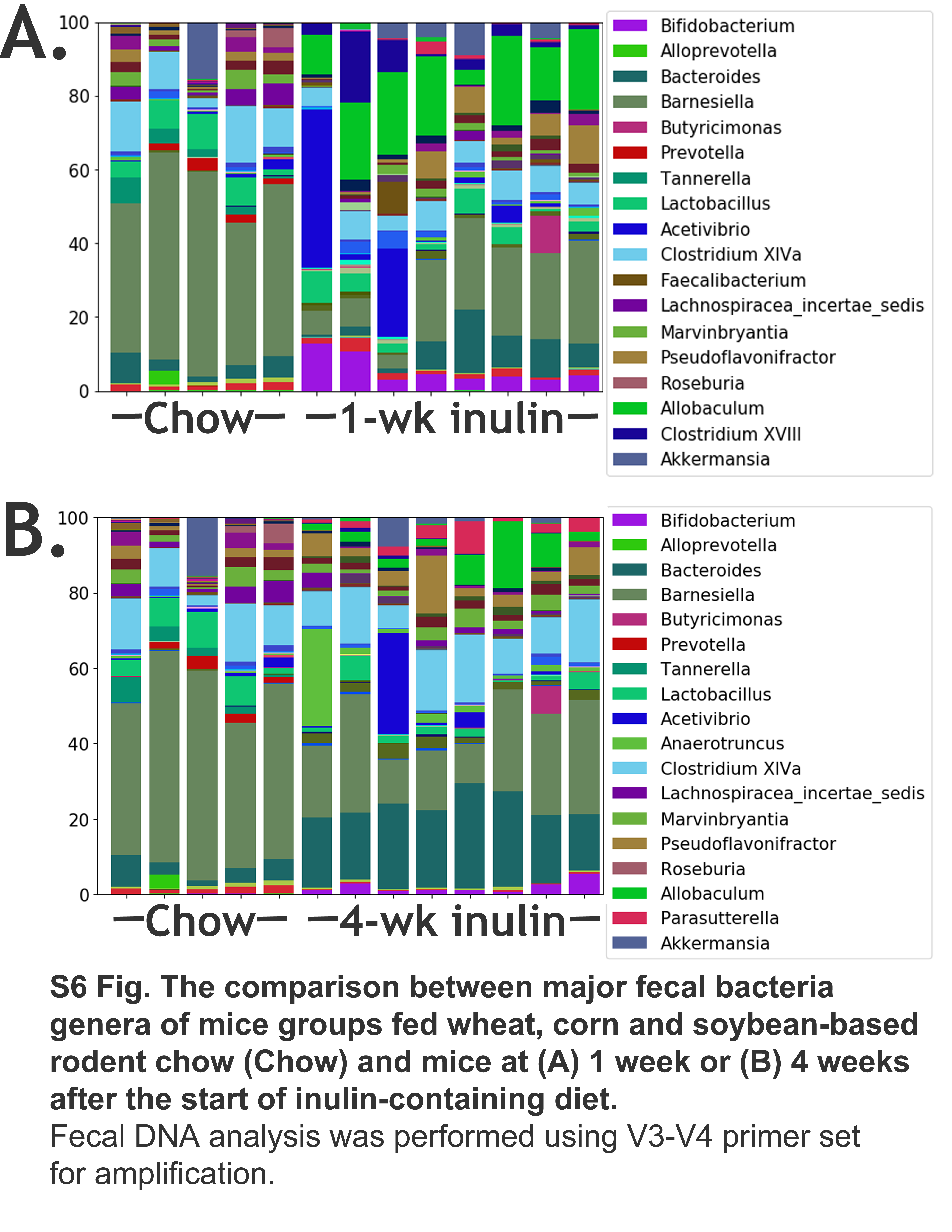

Supplement: S6 Fig — The comparison between major fecal bacteria genera of mice groups fed wheat, corn and soybean-based rodent chow (Chow) and mice at (A) 1 week or (B) 4 weeks after the start of inulin-containing diet. (TIF) [file pone.0205055.s007.tif]
